# Obstructive Sleep Apnea-Hypopnea Syndrome (OSAHS) Combined With Obesity Leads to Elevated Thyroid Hormone Levels

**DOI:** 10.1155/ije/1159707

**Published:** 2025-05-21

**Authors:** Shenjie Xu, Bin Xiang, Lu Ye, Yifeng Jin, Jie Li

**Affiliations:** ^1^Department of General Practice, The First Affiliated Hospital of Soochow University, Suzhou, China; ^2^Department of Respiratory Medicine, Chengdu Sixth People's Hospital, Chengdu, China

**Keywords:** obesity, obstructive sleep apnea-hypopnea syndrome, thyroid function

## Abstract

**Introduction:** Research indicates a strong link between obesity and alterations in thyroid function among patients diagnosed with obstructive sleep apnea-hypopnea syndrome (OSAHS). Our study aims to investigate the thyroid hormone levels in patients with OSAHS combined with obesity. It seeks to elucidate the changes in thyroid hormones and their potential metabolic risks in these patients, thereby further clarifying the role and clinical significance of thyroid function alterations in OSAHS complicated by obesity.

**Methods:** One hundred and thirty-four patients were divided into four groups, including the normal group, the obesity group, the OSAHS with the obesity group, and the OSAHS group. Serum levels of free triiodothyronine (FT3), free thyroxine (FT4), and thyroid-stimulating hormone (TSH) were analyzed using electrochemiluminescence immunoassay. Clinical metabolic parameters (total cholesterol [TC], triglycerides [TG], high-density lipoprotein cholesterol [HDL-C], low-density lipoprotein cholesterol [LDL-C]) and sleep respiratory monitoring indicators (apnea-hypopnea index [AHI], longest duration of sleep apnea [TAmax], oxygen desaturation index [ODI], mean oxygen saturation [M-SaO_2_], and lowest oxygen saturation [L-SaO_2_]) were also recorded.

**Results:** The OSAHS with the obesity group demonstrated elevated FT3, TSH, and ODI levels but lower L-SaO_2_ level than other groups, and the levels of TG and LDL-C were higher than those in the OSAHS group and the normal group. Additionally, TSH level was positively correlated with LDL-C and BMI, but negatively correlated with L-SaO_2_. In the obesity group, FT3, TSH, TG, ODI, and TAmax levels were higher, while L-SaO_2_ and M-SaO_2_ were lower than those in the normal group.

**Conclusions:** Patients with both OSAHS and obesity are at higher risk of developing subclinical hypothyroidism, with LDL-C, BMI, and L-SaO_2_ levels likely contributing to changes in TSH levels.

## 1. Introduction

Obstructive sleep apnea-hypopnea syndrome (OSAHS) is characterized by recurrent instances of partial or complete upper respiratory tract collapse during sleep, resulting in frequent episodes of apnea or hypopnea. The risk factors associated with OSAHS include obesity and hypothyroidism [1, 2]. OSAHS impacts multiple physiological systems, including the respiratory, nervous, endocrine, cardiovascular, urogenital, and others, posing significant harm to affected individuals and severely compromising their quality of life. OSAHS is often found to be coexisting with other conditions such as thyroid dysfunction, diabetes, bone metabolic disorders, polycystic ovary syndrome, and other diseases [3]. In addition, thyroid dysfunction can also lead to OSAHS [2]. A burgeoning corpus of empirical data increasingly substantiates the intimate nexus between OSAHS and thyroid function.

In recent years, a plethora of clinical studies probing into the correlation between OSAHS and thyroid function has produced a spectrum of divergent results. Hypothyroidism has been identified as both a risk factor for OSAHS and a consequence thereof. Certain studies have indicated a notably elevated prevalence of subclinical hypothyroidism among individuals with OSAHS compared to those without the condition [4]. Bahammam et al. discovered that among individuals with OSAHS, the incidence of newly diagnosed subclinical hypothyroidism was 11.1%, while it was 4% among those without OSAHS [5]. This suggests a notable rise in the prevalence of hypothyroidism among OSAHS patients. However, other studies have refuted this finding, demonstrating no significant difference in the prevalence of hypothyroidism between OSAHS patients and normal controls [6]. At present, despite the incongruent conclusions drawn from various studies examining the relationship between OSAHS and thyroid function, a majority of scholars concur that OSAHS has the potential to impact thyroid hormone levels and is intrinsically linked to hypothyroidism.

With the advancement of living standards and the evolution of modern lifestyles, the co-occurrence of OSAHS and obesity has become increasingly prevalent. Among OSAHS patients, the prevalence of obesity is notably elevated. Obesity, an independent risk factor, can result in OSAHS. In addition, hypothyroidism is able to cause weight gain, leading to obesity. At the same time, abnormal thyroid function in obese individuals is not uncommon. In a retrospective examination of obesity and thyroid function, a positive correlation was observed between thyroid-stimulating hormone (TSH) levels and body mass index (BMI) among individuals with normal thyroid function. Additionally, obese individuals exhibited elevated levels of both TSH and free triiodothyronine (FT3) [7]. Unfortunately, a dearth of reports concerning thyroid function in individuals was affected by both OSAHS and obesity. Our objective was to investigate thyroid hormone levels in patients presenting with OSAHS and obesity, aiming to analyze the correlation between thyroid hormone levels and OSAHS, obesity, and other pertinent indicators, with the aim of elucidating factors that potentially influence thyroid function.

## 2. Methods

### 2.1. Research Subjects and Groups

A total of 134 patients were enrolled in the study and subsequently divided into four groups, including the normal group, the obesity group, the OSAHS group, and the OSAHS with the obesity group. Fifty-eight individuals were categorized into the obesity group (38 cases) and the normal group (20 cases) based on the BMI. Seventy-six patients diagnosed with OSAHS via polysomnography (PSG) were stratified into the OSAHS with the obesity group (*n* = 46, BMI ≥ 25 kg/m^2^) and the OSAHS group (*n* = 30, BMI < 25 kg/m^2^) based on the BMI. OSAHS was diagnosed when the apnea-hypopnea index (AHI) was ≥ 5, utilizing PSG (WANMAN SOMNOlab 2). Additional sleep parameters measured included the oxygen desaturation index (ODI), the lowest oxygen saturation (L-SaO_2_), mean oxygen saturation (M-SaO_2_), and the longest duration of sleep apnea (TAmax). The study protocol was approved by the Ethics Committee, and written informed consent was obtained from all participants.

### 2.2. Exclusion Criteria

Exclusion criteria comprised individuals under 18 years of age, those with cancer, heart failure, severe liver or kidney dysfunction, other sleep disorders (such as central sleep apnea or mixed sleep apnea), mental or brain diseases, infectious or rheumatic immune diseases, use of sedative and hypnotic medications, history of thyroid disease or surgical resection of the thyroid gland, and use of medications potentially affecting thyroid hormone levels.

### 2.3. Data Collection

Baseline characteristics such as age, sex, body weight, height, smoking status, and presence of diabetes were documented. Laboratory assessments included measurements of total cholesterol (TC), triglycerides (TG), high-density lipoprotein cholesterol (HDL-C), low-density lipoprotein cholesterol (LDL-C), FT3, FT4, and TSH. Thyroid function was evaluated using electrochemiluminescence immunoassay. The normal reference ranges for TSH, FT3, and FT4 were 0.27–4.20 mIU/L, 3.1–6.8 pmol/L, and 12–22 pmol/L.

### 2.4. Statistical Analysis

Statistical analysis was conducted utilizing SPSS Version 25.0. The data are presented as mean ± standard deviation (SD). Associations between variables and the four groups were assessed using one-way analysis of variance (ANOVA). Subsequently, variables with *p* < 0.05 were subjected to pairwise comparisons using post hoc multiple comparisons and the Bonferroni test. Categorical data were compared using the chi-square test. Pearson correlation analysis was employed to investigate potential correlations between factors. Multivariate linear regression analysis was utilized for multivariate analysis. *p* < 0.05 was considered statistically significant.

## 3. Results

### 3.1. Obesity Is a Risk Factor for Metabolic Abnormalities in the OSAHS Group

Table 1 presents a comparison of metabolic indicators. Significant differences were observed in TC, TG, HDL-C, and LDL-C among the four groups. The results in Table 1 indicated that the TG level was elevated in the obesity group compared to the normal group, while TC, HDL-C, and LDL-C showed no statistically significant differences. In the OSAHS with the obesity group, TC, TG, and LDL-C were significantly higher compared to the normal group. These results suggest that the presence of OSAHS may be associated with lipid abnormalities, particularly when combined with obesity. Furthermore, a comparison between the OSAHS group and the OSAHS with the obesity group indicated that obesity, as a risk factor, can also contribute to increases in TG and LDL-C levels, leading to a decrease in HDL-C. The above results suggest that obesity may exacerbate the negative impact of OSAHS on lipid metabolism. Therefore, we propose that OSAHS itself may have an effect on lipid metabolism, which can be further intensified by obesity.

### 3.2. Obese Individuals Are More Prone to Developing Severe OSAHS

Table 2 presents a comparison of PSG parameters among the four groups. Significant differences were observed in the AHI, ODI, mean oxygen saturation (M-SaO_2_), lowest oxygen saturation (L-SaO_2_), and longest apnea duration (TAmax) among the four groups. When comparing the OSAHS group with the normal group and the obesity group, statistically significant differences were found in all measured parameters (AHI, ODI, M-SaO_2_, L-SaO_2_, and TAmax). The increases in AHI, ODI, and TAmax, along with the decreases in M-SaO_2_ and L-SaO_2_, indicated the occurrence of OSAHS. Compared to the normal group, the obesity group showed significantly higher ODI and TAmax, accompanied by decreases in M-SaO_2_ and L-SaO_2_, suggesting that obese patients were more prone to developing OSAHS. Comparing the OSAHS group with the obesity group revealed that the increases in AHI, ODI, and TAmax were more pronounced in the OSAHS group, with more significant decreases in M-SaO_2_ and L-SaO_2_, indicating that the impact of OSAHS on these parameters was far greater than that of obesity alone. Additionally, the parameters were significantly more abnormal in the OSAHS with the obesity group, indicating that obesity, as one of the risk factors, exacerbated OSAHS.

### 3.3. The Prevalence of Subclinical Hypothyroidism Is Higher in the OSAHS With the Obesity Group

As shown in Table 3, there were nine cases of subclinical hypothyroidism characterized by elevated TSH and normal FT4 levels in the OSAHS with the obesity group, one case in the OSAHS group, and four cases in the obesity group. The prevalence of subclinical hypothyroidism was highest in the OSAHS with the obesity group, with statistically significant differences observed. This result indicated that patients in the OSAHS with the obesity group have a higher prevalence of subclinical hypothyroidism.

### 3.4. The Combination of OSAHS and Obesity Has a Significant Impact on FT3 and TSH Levels

Table 4 compares the levels of FT3, FT4, and TSH among the four groups. FT3 and TSH levels in the obesity group were elevated compared to the normal group. While in the OSAHS group, the level of TSH was also increased compared to the normal group. The simultaneous presence of OSAHS and obesity in patients was associated with significantly elevated FT3 and TSH levels, indicating a significant impact of this combination on TSH.

### 3.5. Changes in FT3 and TSH Levels in the OSAHS With the Obesity Group and Analysis of Related Factors

Table 5 presents the correlation analysis between FT3/TSH and various factors in patients with OSAHS and obesity. FT3 exhibited no significant correlation with age, TC, TG, HDL-C, LDL-C, AHI, M-SaO_2_, L-SaO_2_, Epworth Sleepiness Scale (ESS), ODI, or TAmax. However, FT3 was significantly positively correlated with the BMI. TSH demonstrated no significant correlation with age, TG, HDL-C, AHI, M-SaO_2_, ESS, ODI, or TAmax. However, TSH exhibited significant positive correlations with TC, LDL-C, and BMI. Furthermore, TSH showed a significant negative correlation with L-SaO_2_.

### 3.6. L-SaO_2_, BMI, and LDL-C May Be Risk Factors Associated With Changes in TSH Levels in Patients With OSAHS Combined With Obesity

Table 6 summarizes the results of the multivariate analysis conducted using stepwise regression. In OSAHS with obesity patients, TSH exhibited significant correlations with LDL-C, BMI, and L-SaO_2_. The findings indicated that L-SaO_2_, BMI, and LDL-C might be potential risk factors associated with TSH alterations in patients who had both OSAHS and obesity.

## 4. Discussion

Our study revealed a connection between thyroid disorders and OSAHS/obesity, highlighting the presence of subclinical hypothyroidism within the OSAHS/obesity cohort. Moreover, the prevalence of subclinical hypothyroidism was notably higher among individuals with OSAHS and obesity in comparison to those solely with OSAHS/obesity. Hypothyroidism is characterized by thyroid hormone insufficiency, which poses significant health risks if left untreated, and may potentially lead to severe consequences and even eventual fatality. Hypothyroidism is typically categorized as either clinical or subclinical, representing common pathological conditions associated with thyroid dysfunction. Subclinical hypothyroidism, characterized by low TSH levels and normal free thyroxine, is frequently viewed as an initial indication of thyroid dysfunction. Although clinical hypothyroidism is relatively rare in patients with OSAHS, the prevalence of subclinical hypothyroidism is significant and warrants attention. Hypothyroidism has the potential to directly contribute to OSAHS or influence it indirectly via its impact on metabolic syndrome. Involved mechanisms includes obesity caused by decreased basal metabolic rate, dysregulation of pharyngeal dilatation muscle, depression of respiratory center, and pharyngeal stenosis caused by mucopolysaccharide deposition in the submucosa [5, 8]. In addition, OSAHS also can lead to hypothyroidism, which may be related to nocturnal hypoxemia. Petrone et al. measured morning TSH, FT3, and FT4 in 125 cases of moderate to severe OSAHS and 60 cases of normal sleep at night, and the results showed that 10 (8%) OSAHS patients had subclinical hypothyroidism [9]. The TSH level was notably decreased among patients with subclinical hypothyroidism after continuous positive airway pressure (CPAP) treatment. This implies a potential association between subclinical hypothyroidism and nocturnal hypoxemia. Numerous investigations both domestically and internationally had delved into the prevalence of thyroid disorders and the intricate interplay between thyroid functionality and the severity of OSAHS. Other research showed that TSH was increased in patients with severe OSAHS. At present, the results on OSAHS and thyroid hormone levels are not consistent. Compared with the normal group, our study indicated that TSH in the OSAHS group was increased, while FT3 and FT4 remained unchanged.

There is a strong link between obesity and thyroid function. The prevalence of elevated TSH is higher in obese people than in normal people [10, 11]. In numerous studies examining the relationship between obesity and thyroid function, TSH has been found to be correlated with body weight and BMI. Additionally, TSH, FT3, and FT4 exhibit distinct patterns of change. Michalaki et al. aimed at the relationship between morbid obesity and thyroid function in adults and demonstrated that TSH was also higher in the obese group than in the normal weight group [12]. TSH often shows high values within the normal range or slightly higher than the norm. However, FT3 changes in obese patients are inconsistent. Marzullo et al. revealed that FT3 was decreased in obese people [13]. Gianluca et al. revealed that FT3 remains within the normal range in obese patients [11]. In conclusion, the FT3 level in obese patients remains inconsistent, and so does FT4. In our study, compared with the normal group, the levels of TSH and FT3 in the obesity group were increased, a finding consistent with most current studies, while FT4 had no significant change.

Our study found that in the OSAHS with the obesity group, FT3 and TSH levels were higher than those in the normal group, the obesity group, and the OSAHS group. However, the FT4 level did not differ significantly among these groups. The TSH level in the OSAHS group was higher than that in the normal group, the FT3 and TSH levels in the obesity group were higher than those in the normal group, and the FT3 and TSH levels in the OSAHS and the obesity group were higher than those in the OSAHS group and obesity group. The findings indicated that the thyroid hormone levels in individuals with both OSAHS and obesity are further elevated compared to those with OSAHS alone or obesity alone. Moreover, there is a tendency for TSH and FT3 levels to increase in patients with OSAHS and obesity. The correlation analysis of TSH and FT3 with factors such as age, sex, blood lipids, BMI, and sleep apnea-related indicators revealed that FT3 is positively correlated with the BMI. Additionally, TSH is positively correlated with TC, LDL-C, and BMI, while it is negatively correlated with L-SaO_2_. Further multiple linear regression analysis summarized that the TSH level is correlated with the BMI and L-SaO_2_ in OSAHS with obesity patients. This change may be related to intermittent hypoxia, inflammation, and obesity. It is well-known that adipose tissue of obese patients secretes different amounts of inflammatory cytokines, such as tumor necrosis factor-α (TNF-α), interleukin-1 (IL-1), and interleukin-6 (IL-6), escaping into the systemic circulation and causing systemic symptoms [14]. On the other hand, OSAHS causes oxidative stress due to intermittent hypoxia, leading to inflammatory response, which is also closely related to inflammatory factors. Ajjan et al. had discovered that TSH could enhance the mRNA expression of Na^+^-I^−^-cotransporters, but these inflammatory factors could inhibit the mRNA expression of Na^+^-I^−^-cotransporters induced by TSH [15]. Besides, inflammatory factors can reduce triiodothyronine (T3) and thyroxine (T4) by inhibiting the expression or activity of Na^+^-I^−^-cotransporter and Na^+^-K^+^-ATPase [16, 17]. This, in turn, compensatorily increases the expression of thyrotropin-releasing hormone (TRH) in the paraventricular nucleus and TSH in the pituitary through a negative feedback mechanism, thus leading to increased levels of TSH and FT3. In addition, leptin, a protein playing an important role in energy balance, is synthesized and secreted by adipocytes in obese people [18]. Leptin stimulation can increase TSH [19]. The increased TRH and TSH in OSAHS with obesity are most likely due to the increased circulating leptin [20]. Leptin itself is also capable to stimulate the production of T3 and increase the production of FT3 by promoting the conversion of T4 to T3 [21].

The high conversion rate of T4 to T3 is seen as a defense mechanism that counters fat accumulation by increasing the basal metabolic rate and energy expenditure [22], leading to an increase in the FT3 level of obese patients. Another mechanism to explain the elevated FT3 levels is related to the lower levels of TSH and thyroid hormones in adipocytes of obese individuals compared with those in normal weight individuals. This will lead to a decrease in tissue responsiveness to circulating thyroid hormones, which in turn will trigger a compensatory increase in the secretion of TSH and FT3, in an attempt to overcome peripheral resistance [23]. Therefore, the elevated levels of FT3 and TSH in patients with OSAHS and obesity, as well as the correlation of TSH with BMI and L-SaO_2_, may primarily be attributed to the combined effects of leptin and inflammation resulting from oxidative stress. In conclusion, the changes in thyroid function observed in these patients may be due to the synergistic impact of OSAHS and obesity. These changes may also be associated with altered TSH bioactivity and an adaptive process that increases resting energy expenditure [22, 24].

Hypothyroidism is not common in OSAHS patients, so it is not advocated to routinely evaluate the thyroid function of suspected OSAHS patients [5]. However, some articles have demonstrated that hypothyroidism in OSAHS patients is not uncommon. Moreover, thyroid surgery significantly improves OSAHS symptoms. Therefore, we recommend routine thyroid function assessment for OSAHS patients [25, 26]. If patients with sleep disorders are not routinely evaluated for thyroid function, it may lead to missed diagnosis and misdiagnosis, potentially compromising the effective treatment of OSAHS. This also increases the risk of complications associated with undiagnosed hypothyroidism, thereby increasing the personal and societal management costs for patients. Sakellaropoulou et al. believed that screening for thyroid function is necessary in children with obesity and sleep apnea [27]. Therefore, assessing TSH levels in obese patients may help rule out possible impairments in resting energy expenditure due to reduced peripheral effects of thyroid hormones.

This study still has some limitations. Regarding thyroid function in patients with OSAHS and obesity, the severity of OSAHS and obesity was not further stratified due to the limited sample size. In the future, efforts will be made to increase the sample size to conduct more detailed severity stratification research, thereby enhancing the accuracy of the results. Additionally, while the changes in thyroid function in patients with OSAHS and obesity may be related to factors such as oxidative stress, inflammatory factors, and leptin, the underlying mechanisms of these relationships have not yet been thoroughly explored. Further research into these mechanisms is warranted.

In conclusion, clinicians are advised to consider the presence of OSAHS and obesity when assessing thyroid dysfunction. In patients with OSAHS and obesity, thyroid function changes are observed, with TSH levels tending to increase and a higher likelihood of subclinical hypothyroidism. Therefore, routine thyroid function testing is recommended for these patients. These thyroid hormone changes might be caused by obesity and OSAHS. Therefore, we can initiate treatment from an etiological perspective, specifically targeting the management of obesity or OSAHS, rather than resorting to indiscriminate thyroid disease treatment. This approach not only addresses the root causes but also provides a new theoretical foundation and data support for the prevention and treatment of thyroid dysfunction in patients with OSAHS and obesity.

## 5. Conclusions

The likelihood of subclinical hypothyroidism increases in patients with OSAHS and obesity, with LDL-C, BMI, and L-SaO_2_ as potential risk factors for elevated TSH levels.

## Figures and Tables

**Table 1:** Comparison of metabolic indexes among the four groups.

	Normal *N* = 20	Obesity *N* = 38	OSAHS *N* = 30	OSAHS with obesity *N* = 46	*F*	*p*
TC ($\overset{¯}{x}$ ± *s*, mmol/L)	4.0 ± 0.8	4.7 ± 1.0	4.5 ± 1.0	5.0 ± 0.9^a^	4.142	0.008
TG ($\overset{¯}{x}$ ± *s*, mmol/L)	1.2 ± 0.5	1.7 ± 0.5^a^	1.5 ± 0.8	2.0 ± 0.7^ab^	9.498	< 0.001
HDL-C ($\overset{¯}{x}$ ± *s*, mmol/L)	1.0 ± 0.2	1.0 ± 0.2	1.1 ± 0.3	0.9 ± 0.2^b^	4.248	0.007
LDL-C ($\overset{¯}{x}$ ± *s*, mmol/L)	2.4 ± 0.6	3.0 ± 1.1	2.5 ± 0.7	3.3 ± 0.9^ab^	7.355	< 0.001

*Note:* TG, triglyceride.

Abbreviations: HDL-C, high-density lipoprotein cholesterol; LDL-C, low-density lipoprotein cholesterol; TC, total cholesterol.

^a^ *p* < 0.05 vs. normal.

^b^ *p* < 0.05 vs. OSAHS.

**Table 2:** Comparison of PSG-related indicators among the four groups.

	Normal *N* = 20	Obesity *N* = 38	OSAHS *N* = 30	OSAHS with obesity *N* = 46	*F*	*p*
AHI (events/h)	1.2 ± 0.6	2.7 ± 0.9	24.8 ± 6.2^ab^	36.5 ± 17.3^abc^	100.839	< 0.01
ODI (events/h)	3.4 ± 2.0	17.4 ± 9.1^a^	49.1 ± 23.1^ab^	54.5 ± 21.5^abc^	60.571	< 0.01
M-SaO_2_ (%)	98.5 ± 1.0	96.8 ± 3.5^a^	95.1 ± 2.1^ab^	93.3 ± 2.4^ab^	35.228	< 0.01
L-SaO_2_ (%)	94.3 ± 3.5	88.2 ± 1.5^a^	79.4 ± 8.5^ab^	69.6 ± 6.3^abc^	120.696	< 0.01
TAmax (seconds)	4.3 ± 2.6	20.6 ± 8.0^a^	45.7 ± 23.3^ab^	56.2 ± 24.7^ab^	48.185	< 0.01

*Note:* Data are means ± SD. L-SaO_2_, lowest oxygen saturation; M-SaO_2_, average oxygen saturation; TAmax, the longest time of apnea.

Abbreviations: AHI, apnea-hypopnea index; ODI, oxygen desaturation index.

^a^ *p* < 0.05 vs. normal.

^b^ *p* < 0.05 vs. obesity.

^c^ *p* < 0.05 vs. OSAHS.

**Table 3:** Comparison of the incidence of subclinical hypothyroidism among the four groups.

	Normal *N* = 20	Obesity *N* = 38	OSAHS *N* = 30	OSAHS with obesity *N* = 46	*χ* ^2^	*p*
Subclinical hypothyroidism (*N* and [%])	0 (0)	4 (10.5)	1 (3.3)	9 (19.6)^abc^	8.044	0.045

^a^ *p* < 0.05 vs. normal.

^b^ *p* < 0.05 vs. obesity.

^c^ *p* < 0.05 vs. OSAHS.

**Table 4:** The level of FT3, FT4, and TSH among the four groups.

	Normal *N* = 20	Obesity *N* = 38	OSAHS *N* = 30	OSAHS with obesity *N* = 46	*F*	*p*
FT3 (pmol/L)	4.2 ± 0.5	4.9 ± 0.7^a^	4.4 ± 0.3	5.4 ± 0.9^abc^	17.446	< 0.001
FT4 (pmol/L)	17.2 ± 1.6	16.1 ± 2.4	16.9 ± 1.8	16.3 ± 2.6	1.569	0.200
TSH (mIU/L)	1.6 ± 0.9	2.6 ± 1.2^a^	2.7 ± 0.5^a^	3.5 ± 1.1^abc^	17.623	< 0.001

*Note:* Data are means ± SD. FT3, free triiodothyronine; FT4, free thyroxine.

Abbreviation: TSH, thyroid-stimulating hormone.

^a^ *p* < 0.05 vs. normal.

^b^ *p* < 0.05 vs. obesity.

^c^ *p* < 0.05 vs. OSAHS.

**Table 5:** Correlation between FT3/TSH and multiple variables.

	FT3		TSH	
	*r*	*p*	*r*	*p*
Age	−0.142	0.347	−0.185	0.129
TC	0.201	0.182	0.303	0.041^∗^
TG	0.145	0.336	0.275	0.064
HDL-C	−0.214	0.154	0.090	0.552
LDL-C	0.141	0.349	0.299	0.043^∗^
AHI	0.048	0.750	0.038	0.800
M-SaO_2_	−0.114	0.452	0.162	0.281
L-SaO_2_	0.036	0.811	−0.450	0.002^∗^
ESS	0.086	0.568	0.196	0.191
TAmax	−0.152	0.313	−0.256	0.086
BMI	0.433	0.003^∗^	0.374	0.010^∗^
ODI	−0.114	0.452	0.162	0.281

*Note:* Each digit expresses a correlation coefficient.

^∗^ *p* < 0.05.

**Table 6:** Stepwise regression analysis of TSH and related indicators.

Model		*β*	Beta	*t*	*p*
1	(Constant)	8.706		5.521	< 0.001
	L-SaO_2_	−0.076	−0.450	−3.346	0.002

2	(Constant)	6.940		4.525	< 0.001
	L-SaO_2_	−0.078	−0.467	−3.817	< 0.001
	BMI	0.047	0.394	3.218	0.002

3	(Constant)	5.459		3.342	0.002
	L-SaO_2_	−0.071	−0.425	−3.565	0.001
	BMI	0.049	0.407	3.451	0.001
	LDL-C	0.283	0.252	2.111	0.041

*Note:* Model 1: *R*^2^ = 0.203, *F* = 11.198, and *p*=0.002; Model 2: *R*^2^ = 0.358, *F* = 11.966, and *p* < 0.001; Model 3: *R*^2^ = 0.419, *F* = 10.104, and *p* < 0.001.

## Data Availability

The data that support the findings of this study are available from the corresponding authors upon reasonable request.

## Supporting Information

The BMI and ESS were highest in the OSAHS with the obesity group.

As presented in Table S1, a total of 134 participants, comprising 65 males and 69 females, were included in the study, with their ages ranging from 21 to 75 years. Notably, there were no significant differences among all groups in terms of age, gender, smoking status, hypertension, and diabetes. BMI and ESS were significantly different among the four groups. Differences in BMI and ESS were observed among all groups, showing a gradient increase. The findings revealed that obesity and OSAHS significantly impacted sleepiness, with increasing weight correlating to greater severity of OSAHS. Moreover, when these two conditions coexisted, the increases in these indices were even more pronounced.

Table S1: Baseline characteristics.
