# Supplementary material for: Obstructive Sleep Apnea-Hypopnea Syndrome (OSAHS) Combined With Obesity Leads to Elevated Thyroid Hormone Levels
Source: Int J Endocrinol. 2025 May 21;2025:1159707. doi: 10.1155/ije/1159707 (PMC12119152; doi:10.1155/ije/1159707)
Supplement: Supporting Information — Additional supporting information can be found online in the Supporting Information section. [file 1159707.f1.docx]

**Table S1** Baseline characteristics

|  | Normal  N=20 | Obesity  N=38 | OSAHS  N=30 | OSAHS with Obesity  N=46 | *F*/χ^2^ | *P* |
| --- | --- | --- | --- | --- | --- | --- |
| Age（‾x±s,years） | 46.3±10.1 | 48.4±12.5 | 49.2±11.5 | 49.1±12.5 | 0.305 | 0.822 |
| Sex（male/female） | 11/9 | 13/25 | 19/11 | 22/24 | 6.096 | 0.107 |
| Smoking  [N and (%)] | 3（15） | 4（10.5） | 5（16.7） | 10（21.7） | 1.941 | 0.585 |
| Hypertension  [N and (%)] | 5（25） | 6（15.8） | 10（33.3） | 14（30.4） | 3.367 | 0.338 |
| Diabetes  [N and (%)] | 3（15） | 10（26.3） | 5（16.7） | 12（26.1） | 1.893 | 0.595 |
| BMI（kg/m^2^,‾x±s) | 19.3±0.7 | 36.3±4.6^a^ | 24.0±0.5^ab^ | 41.7±8.8^abc^ | 102.319 | ＜0.001 |
| ESS（score,‾x±s） | 3.8±2.2 | 4.3±2.7^a^ | 10.2±1.3^ab^ | 14.0±2.3^abc^ | 187.797 | ＜0.01 |

^a^ *P* < 0.05 vs Normal; ^b^ *P* < 0.05 vs Obesity; ^c^ *P* < 0.05 vs OSAHS

BMI*,* body mass index; ESS*,* Epworth sleepiness score.
